# Chemotherapy Associated Ovarian Failure

**DOI:** 10.3389/fendo.2020.572388

**Published:** 2020-12-08

**Authors:** Davide Mauri, Ioanna Gazouli, Georgios Zarkavelis, Alexandra Papadaki, Leonidas Mavroeidis, Stefania Gkoura, Panagiotis Ntellas, Anna-Lea Amylidi, Lampriani Tsali, Eleftherios Kampletsas

**Affiliations:** ^1^ Faculty of Medicine, School of Health Sciences, University of Ioannina, Ioannina, Greece; ^2^ Department of Medical, Oncology, Greece Society for Study of Clonal Heterogeneity of Neoplasia (EMEKEN), University Hospital of Ioannina, Ioannina, Greece; ^3^ Associated Scientific Partner, Athens, Greece

**Keywords:** ovarian reserve, ovarian failure, sterility, cancer, chemotherapy

## Abstract

As the incidence of malignancies in young adults is increasing, fertility preservation in cancer survivors arises as a major concern. Especially among female cancer patients, pregnancy rates are estimated to be 40% lower compared to women of the same age. Nowadays oncologists are to be preoccupied not only with their patients’ successful treatment, but also with the maintenance of the potential of the latter to conceive and obtain children. Chemotherapy associated ovarian failure (COF), refers to disruption of ovarian function both as an endocrine gland and as a reproductive organ, due to previous exposure to chemotherapy agents. Although the underlying mechanism is not fully understood, it is supposed that chemotherapy agents may induce either DNA damage of premature ovarian follicle or early activation and apoptosis of them, resulting into early exhaustion of available follicle deposit. Various chemotherapy agents have been associated with COF with the highest incidence being reported for patients undergoing combination regimens. Although a variety of alternatives in order to maintain ovarian function and fertility in female cancer survivors are available, adequately established practices to do so are lacking. Thus, it is of major importance to investigate further and collect sufficient evidence, aiming to guide patients and physicians in everyday clinical practice.

## Introduction

Over 6.6 million women are estimated to be annually diagnosed with cancer, about 10% of them being younger than 40 years old (1). On the other hand, in modern Western societies, an increasing proportion of women delay their first pregnancy until the fourth decade of life (2). Notably, female cancer survivors are 40% less probable to become pregnant, compared to healthy women, with low pregnancy rates mainly reported among patients diagnosed with leukemia, cervical and breast cancer (3, 4). In this context, nowadays oncologists are not only to be preoccupied with their patients’ successful treatment, but also with major fertility preservation concerns. In the following paragraphs, we attempt to summarize the underlying mechanisms of COF, as well as the current therapeutic and preventive strategies addressing female fertility maintenance dilemmas.

## COF—Definition and Associated Antineoplastic Agents

Chemotherapy associated ovarian failure (COF) refers to disruption of both endocrine and reproductive ovarian function, after exposure to chemotherapy. It is defined as either the absence of regular menses in premenopausal female patients or as increased FSH levels (>40 IU/L) (5).

In 2006, the American Association of clinical oncology attempted to sort antineoplastic regimens, according to the associated fertility compromise risk. Hematopoietic stem cell transplant (HSCT) initiation regimens steadily compromise patients’ fertility, while gonadotoxicity of adjuvant chemotherapy regimens against early breast cancer varies with duration of exposure and patient’s age. Characteristically, triple agent combinations, such as CMF (cyclophosphamide, methotrexate, fluorouracil), entail a high risk of infertility if administered for more than four cycles in women older than 40, whereas the risk is significantly reduced for younger patients. Notably, vincristine, methotrexate, and fluorouracil do not impose considerable fertility hazards, while there are no sufficient date regarding taxanes, oxaliplatin, and targeted treatments (6) (**Table 1**).

**Table 1:** Risk of infertility associated with antineoplastic systematic treatment [based on American Society of Clinical Oncology Recommendations on Fertility Preservation in Cancer Patients, (6).

Risk category	Related malignancies	Chemotherapy regimens	Patients age
**High risk >80%**	Various hematologic malignancies or solid tumours	HSCT initiation including cyclophosphamide/total body irradiation or cyclophosphamide/busulfan	NR
	Adjuvant early breast cancer chemotherapy	CMF, CEF, CAF ×6 or more cycles	40 yrs or older
**Intermediate risk 20–80%**	Adjuvant early breast cancer chemotherapy	CMF, CEF, CAF ×6 or more cycles	30 to 39 yrs
		AC ×4 cycles	40 yrs or older
**Low risk <20%**	Non-Hodgkin lymphoma	CHOP ×4–6 cycles, CVP	NR
	Hodgkin lymphoma	ABVD ×4–6 cycles	
	Acute myeloid leukemia	Anthracycline and cytarabine	
	Acute lymphocytic leukemia	Multi-agent	
	Adjuvant early breast cancer chemotherapy	CMF, CEF, CAF ×6	30 yrs or younger
		AC ×4	40 yrs or younger
**Very low or low risk**	Germ cell tumors, GI tumors	Vincristine, Methotrexate, Fluorouracil	–
**Unknown**	GI tumors, breast cancer, lung cancer	Taxanes Oxaliplatin Irinotecan	–
	GI tumors, breast cancer, melanoma, lung cancer	Monoclonal antibodies (trastuzumab, bevacizumab, cetuximab) Tyrosine kinase inhibitors	–

HSCT, hematopoietic stem cell transplant; NR, not reported; CMF, cyclophosphamide, methotrexate, fluorouracil; CEF, cyclophosphamide, epirubicin, fluorouracil; CAF, cyclophosphamide, doxorubicin, fluorouracil; AC, doxorubicin, cyclophosphamide; ABVD, doxorubicin, bleomycin, vinblastine, dacarbazine; CHOP, cyclophosphamide, doxorubicin, vincristine, prednisone; CVP, cyclophosphamide, vincristine, prednisone.

Considering the finite number of follicles available in the ovaries and their co-existence in different stages of development, variable pathophysiologic mechanisms have been proposed to underlie chemotherapy induced ovarian failure (see **Table 2**). These include:

- “Accelerated” ovarian follicle maturation: Chemotherapy agents induce apoptosis of mature, functioning ovarian follicles, resulting in depression of estrogen and anti-müllerian hormone negative feedback on the gonadotropic cells of the anterior pituitary. Constantly elevated gonadotropins may accelerate maturation of premature ovarian follicles, which, in their turn, enter apoptosis under systematic chemotherapy, thus the gradual exhaustion of ovarian follicles deposit (5, 7, 8). Supporting evidence comes from histology studies of murine ovarian tissue, in cyclophosphamide treated mice, showing increased population of early growing follicles, in parallel with elimination of the quiescent ones (8). The enhanced phosphorylation of proteins involved in the maturation of primordial follicles seems to be mediated *via* the PI3K/PTEN/Akt signaling pathway, which may also be activated due to a direct effect of chemotherapy on oocytes and on pregranulosa cells supporting them (7–9).
- Direct quiescent follicle DNA damage: Non-cell cycle specific chemotherapeutics, such as alkylating agents and doxorubicin, can induce formation of cross-links in the DNA of non-dividing, dormant oocytes. The subsequent accumulation of DNA strand breaks activates the pro-apoptotic intracellular pathways, leading to apoptosis of the affected ovarian follicles (10). Relevant supporting evidence derives from studies of human oocyte *in vitro* cultures and human ovarian xenograft murine models, exposed to doxorubicin (11) and cyclophosphamide (12), revealing double strand breaks and features of apoptotic death in premature oocytes.
- Disrupted ovarian vascularization: Chemotherapy may compromise the functionality of ovarian vasculature and stroma supporting the gonadal cells. Local vascular spasm reducing ovarian blood flow, fibrosis of the ovarian cortex affecting blood vessel formation, inhibition of angiogenesis, are some of the described associated mechanisms. Relative evidence has been found in *in vitro* and murine xenograft studies of human ovarian tissue, as well as mouse ovaries, exposed to doxorubicin (10, 13).

**Table 2:** Summary of the suggested underlying mechanisms by which chemotherapy compromises follicular ovarian reserve (5, 7–13).

Proposed mechanism	Outline	Reference
“Accelerated” ovarian follicle maturation	Chemotherapy→ apoptosis of functioning ovarian follicles→ ↓estrogen, anti-Müllerian hormone→ ↑ gonadotropins → accelerated maturation of premature ovarian follicles→ apoptosis → gradual exhaustion of ovarian follicles deposit	Cui et al. (5) Roness et al. (7) Kalich-Philosoph et al. (8)
	Chemotherapy → ↑ activation of the PI3K/PTEN/Akt signaling pathway in oocytes and pregranulosa cells → phosphorylation of maturation mediators → apoptosis of mature follicles → gradual exhaustion of ovarian follicles deposit	Roness et al. (7) Kalich-Philosoph et al. (8) Adhikari et al. (9)
Direct quiescent follicle DNA damage	Alkylating agents, doxorubicin→ formation of DNA cross-links in non-dividing, dormant oocytes→ accumulation of DNA strand breaks→ pro-apoptotic intracellular pathways activation→direct apoptosis of quiescent follicles→ gradual exhaustion of ovarian follicles deposit	Bedoschi et al. (10) Soleimani et al. (11) Titus et al. (12)
Disrupted ovarian vascularization	Chemotherapy→ ovarian vascular spasm → ovarian ischemia related damage	Bedoschi et al. (10) Bar-Joseph et al. (13)
	Chemotherapy→fibrosis of the ovarian cortex→ compromised blood vessel formation→ ovarian ischemia related damage	
	Chemotherapy→ inhibition of angiogenesis → reduced ovarian blood flow→ ovarian ischemia related damage	

## Ovarian Function Preservation Approaches

### GnRH Analogs: Attempting to Block Premature Follicle Activation

Constant GnRH analogs administration during chemotherapy has been thought to inhibit early ovarian follicle recruitment, by desensitizing hypophysis to the innate GnRH effect (7). GnRH analogs have been mostly employed for fertility preservation in early breast cancer and lymphoma female patients, with ambiguous results.

About 20.038 women aged between 15 and 44 years are yearly diagnosed with early breast cancer in the US, and 97% of them face a risk of infertility due to adjuvant chemotherapy, while half of them wish to have children (14). The potential protective effect of GnRHa administration concurrently with adjuvant chemotherapy, in order to protect ovarian function, has been addressed in several clinical trials (15–17).

A metanalysis of seven placebo-controlled, randomized clinical trials, recruiting 1,047 patients, conducted between 1975 and 2015 seems to favor GnRH administration (15). GnRH analogs employed included goserelin (three trials), triptorelin (three trials), and leuprolide (one trial), while chemotherapy consisted mainly of anthracyclines, cyclophosphamide, and taxanes. Use of tamoxifen was reported in six trials (0% in three trials and about 70% in another three trials). GnRHa administration seemed to double rates of regular menstruation, compared to placebo, at 6 (OR = 2.41, 95% CI 1.40–4.15, p = 0.002) and 12 months (OR = 1.85, 95% CI 1.33–2.59, p = 0.0003) after chemotherapy withdrawal. Patients on GnRHa during adjuvant chemotherapy also seemed to have almost twice the chance of pregnancy, compared to the untreated women (OR 1.85, 95% IC 1.02–3.36, p = 0.04) (15, 17).

In a more recent report of the PROMISE-GIM6 trial, included in the above metanalysis, neither recovery of menstruation (HR of 1.28, 95% CI 0.98–1.68, p = 0.071) nor pregnancy rates (2.56, 95% CI 0.68–9.6, p = 0.142) were found significantly higher in triptorelin treated patients, at 7 years of follow-up (18). Nonetheless, patients’ age may act as a confounding factor; in the OPTION trial (19), goserelin administration conferred an advantage in patients younger than 40 years old, as COF was observed at 2.6% of goserelin treated patients and at 20% (p = 0.038) of placebo treated patients, while no benefit was established in the total trial population (COF incidence in 18.5 *vs.* 34.8% in the goserelin and control arm, respectively, p = 0.048).

Hodgkin and non-Hodgkin lymphomas are estimated to affect up to 18 women aged between 15 and 39 years old, per 100.000 of population (20, 21). A metanalysis of three randomized clinical trials and four case-control series, including a total of 434 lymphoma patients under systematic chemotherapy, deduced that GnRHa treatment seemed to decrease the incidence of COF, defined as increased FSH level, by 68% (OR 0.32 95% CI, 0.13–0.77, p = 0.01) (22). In contrast, spontaneous pregnancy rates were not significantly affected with 13.5 and 11% of survivors getting pregnant in GnRHa and placebo treated groups, respectively (OR = 1.11, 95% CI 0.55–2.26, p = 0.75). Nevertheless, the most recent, randomized, multicenter clinical trial addressing COF in female patients having undergone chemotherapy for Hodgkin or non-Hodgkin lymphomas suggests otherwise (23). Among 67 evaluable lymphoma female patients treated with alkylating agents between 2002 and 2008, COF (defined as at least one measurement of FSH level >40 IU/L) occurred in 19.5 and 25% of patients in the triptorelin and control arm respectively. Triptorelin administration was not an independent prognostic factor for patient protection from COF, in the multivariate analysis (OR = 0.7, 95% CI 0.15–3.24, p = 0.651); in this trial, occurrence of COF after chemotherapy was found to be increased 70-fold after an initiation regimen for hematopoietic stem cell transplant, and 10-fold by administration of a cumulative dose of cyclophosphamide greater than 5 g/m2. In addition, both groups achieved similar pregnancy rates (53% in the triptorelin treated patients, and 43% in the placebo, p = 0.467), three pregnancies occurring among placebo treated women diagnosed with protocol defined COF.

There is only one small, randomized clinical trial addressing the effectiveness of GnRHa in patients treated for ovarian cancer, with conservative surgery and adjuvant chemotherapy. Thirty patients aged between 12 and 45 years, among whom 20 were diagnosed with germ cell tumors, were 1:1 randomized to receive the GnRHa diphereline or nothing, during chemotherapy. Employed regimens included BEP (bleomycin, etoposide, cisplatin) (13 in GnRHa arm/9 in control arm), Carboplatin plus paclitaxel (2 in GnRHa arm/4 in control arm), cisplatin plus paclitaxel (0 in GnRHa arm/1 in control arm), VAC (0 in GnRHa arm/1 in control arm). COF was defined as permanent absence of menses and FSH higher than 20 mIU/ml at 6 months after chemotherapy completion. All patients receiving diphereline experienced recovery of menses, and had premenopausal FSH and estradiol values, whereas one third of patients in the control group had permanent cessation of menses, high FSH, and low estradiol levels. Remarkably, cyclophosphamide and cisplatin, the two most gonadotoxic agents, were administered only in two patients, both of them in the control group, while it is not reported if these two patients were among the five ones experiencing permanent COF (24).

Moreover, in a small study pre- and post-menarchal patients treated for Hodgkin and non-Hodgkin lymphoma, thymoma, acute myeloid, and lymphoid leukemia, GnRH treatment confined a more notable benefit in preserving menstruation and fertility in postpubertal patients, whereas prepubertal girls seemed to be at less risk of COF, even in the absence of GnRH treatment (25).

In conclusion, GnRH analog treatment is not adequately established and it is not currently suggested as a reliable measure of fertility preservation by international guidelines, although it appears to have some protective effect, especially in younger patients. More studies and more long-term results of the already conducted trials are needed to further investigate this question.

### Oocyte/Embryo Cryopreservation

Oocyte or embryo cryopreservation may be recommended to premenopausal women affected by any type of malignancy (4). Oocyte cryopreservation is performed by ovarian hyperstimulation by gonadotropins and freezing of the transvaginally retrieved mature oocytes. The embryo cryopreservation protocols include *in vitro* insemination of the collected oocytes before storage. When conception is desired, either defrosted *in vitro* fertilized oocytes or defrosted embryos are introduced in the patient (26). Little is known about the potential of the ovarian stimulation to promote growth of hormone-driven neoplasms, suggesting that this strategy should probably be withheld for aggressive and hormone sensitive disease (4, 27).

The oocyte cryopreservation protocol (26) begins with controlled ovarian stimulation of the patient, by administration of FSH, follitropin alpha, lutropin alfa, and urofolitropin, starting at 2–3 days after the onset of menstruation. Mature oocytes then transvaginally collected under ultrasound guidance, after hCG administration. Oocyte insemination for embryo preservation is achieved *via in vitro* intracytoplasmic sperm injection (ICSI). Oocytes or embryos are then exposed to an ethyl glycol and dimethylsulphoxide solution and inserted in storage straws, within which they are frozen by immersion in liquid nitrogen.

Frozen eggs and embryos can be rewarmed by insertion in culture dishes, within sucrose-based culture media. Next, *in vitro* fertilized oocytes or embryos are re-introduced in the patient, after sufficient preparation with systematic and transvaginal estradiol administration.

Encouragingly, frozen oocytes are equally prone to *in vitro* fertilization compared to fresh ones (70 *vs* 72%), and even more fruitful considering embryo implantation rates (43 *vs* 35%) as well as clinical pregnancies achieved per transfer (57 *vs* 44%) (28). Besides, among 900 children born by 2009, employing cryopreservation methods, congenital anomalies rate did not differ significantly from the general population (29). However, the effectiveness of cryopreservation among female cancer survivors has not been systematically recorded (30). In a retrospective trial, performed in a tertiary care referral center, only 11 of 252 premenopausal female cancer patients attempted fertilization after cancer remission, four of them achieving pregnancies, and two ending up with a healthy delivery (31). Accordingly, oncologic female patients tend to accomplish lower implantation rates (32.5 *vs* 42.6%) as well as fewer pregnancies (35.7 *vs* 57.7%) and live deliveries (41.1 *vs* 68.8%) compared to age matched controls. In spite of these limitations, oocyte/embryo cryopreservation in cancer patients should be encouraged, as they may offer the patient a fair chance of preserving their fertility (32).

### Cryopreservation of Ovarian Tissue

Cryopreservation of ovarian tissue, aspires to fully recover the ovarian endocrine and reproductive function, after being re-transplanted to the patient. Markedly, it is applicable to prepubescent girls, while not requiring potentially harmful hormonal pretreatment (33).

Indeed, 130 live births have been described worldwide, resulting from transplantation of cryopreserved ovarian tissue (33). Normal ovarian function is restored in 64% of patients undergoing autotransplantation, 58% of them achieving uncomplicated childbearing and delivery (34).

The procedure consists of laparoscopic ovariectomy, followed by dissection and vitrification of the obtained ovarian tissue (35). When restoration of the ovarian reproductive function is desired, vitrificated ovarian tissue is warmed, inoculated *in vitro* with Akt stimulators, and laparoscopically inserted in the subserosa of the fallopian tubes. After ultrasonographic confirmation of follicle maturation, the latter are transvaginally collected, *in vitro* fertilized, and re-introduced to the patient (35). Unfortunately, there are no valid biomarkers to assess the residual follicles deposit in the preserved tissue, in order to predict the expected patient’s potential to produce mature follicles (33, 35).

A key question about cryopreservation is the establishment of an optimal freezing protocol, as too slow and too rapid freezing procedures may cause osmotic cell dehydration and intracellular water crystal formation, respectively, both being detrimental to the ovarian tissue. Thus, most protocols include the use of cryoprotectants, such as glycerol, DMSO, and ethylene glycol, although at high concentrations such substances also exert a toxic effect on the ovarian tissue, creating another concern for clinical practice (33).

Vitrification is an alternative cryopreservation method consisting in the conversion of the resected ovarian tissue to a preservable glass-like solid, by ultrafast cooling in the presence of high levels of cryoprotectants (33). Despite appearing as a promising choice it has not been adequately evaluated in clinical practice. In a series of 37 patients undergoing vitrification for primary ovarian insufficiency (POI), published in 2015, IVF and embryo transfer were finally performed in four of them, resulting in three pregnancies, two of which leading to live births and one ending up with a miscarriage (35). In an earlier series of 27 POI patients, one live delivery was noted, among three patients undergoing IVF and embryo transfer (36).

In conclusion, cryopreservation of ovarian tissue is an alternative solution for fertility preservation, applicable to prepubertal patients, which should be further investigated, in order to overcome technical obstacles and obtain relevant clinical experience (33, 35).

## Alternative Therapeutic Approaches—Preclinical Data

Except from the GnRH analogs, other pharmaceutical agents have been explored in the preclinical setting within the last 20 years, in the context of fertility preservation (see **Table 3**). These include:

**Table 3:** Summary of the alternative therapeutic agents under preclinical investigation (7, 20, 37–42).

Investigated alternative agent	Mechanism of action	Potential drawbacks	Reference
S1P	Inhibition of sphingomyelinase → reduced hydrolysis of the cell membrane lipids→reduction of the pro-apoptotic molecule ceramide→ limitation of primordial follicles cell death	S1P anti-apoptotic effect may antagonize the cytotoxicity of chemotherapy agents	Morita et al. (37)
Imatinib	Inhibition of c-ABl kinase → apoptotic pathway blockade in primordial follicles	Results not replicated in more recent experiments	Gonfloni et al. (38) Kerr et al. (39)
AS101	Reduced activation of the PI3K/PTEN/Akt pathway→reduced primordial follicle maturation →reduced accelerated maturation and death of quiescent follicles	Not reported—actually it may exert an anti-tumor effect	Eichenauer et al. (20) Carmely et al. (40)
G-CSF	↑neovascularization of the ovarian tissue→ protection from ischemia	Not reported	Skaznik-Wikiel et al. (41)
Tamoxifen	Estrogen antagonist- potentially up-regulates IGF-1→ protection of primordial follicles from oxidative stress	Not reported	Roness et al. (7) Ting et al. (42)

S1P, sphingosine-1-phosphate; AS101, ammonium trichloro(dioxoethylene-o,o′)tellurate; G-CSF, Granulocyte colony-stimulating factor; IGF-1, Insulin-like Growth Factor 1.

Sphingosine-1-phosphate (S1P): The sphingomyelinase pathway may mediate the activation of cell death in primordial follicles, *via* accumulation of ceramide, an apoptotic molecular messenger, produced by sphingomyelinase catalyzed hydrolysis of the cellular membrane. Indeed, murine oocytes in which the sphingomyelinase gene has been either knocked down or inhibited by the molecule S1P resisted normal developmental apoptosis during gametogenesis. Similarly, in murine models treated with S1P, primordial ovarian follicles also resisted radiation induced apoptosis. Consequently, S1P may be a promising agent to be further investigated in future studies, although its anti-apoptotic effect may potentially compromise the cytotoxicity of chemotherapy agents (37).

Imatinib: a widely used tyrosine kinase inhibitor, has been thought to exert an anti-apoptotic effect in primordial ovarian follicles, through inhibition of c-ABl kinase mediated apoptotic pathway. Imatinib co-administration with cisplatin to rodent models can limit death of primordial follicles, preserving reproductive ovarian function (38), although these results were not replicated (39).

AS101: AS101 acts as a modulator of the PI3K/PTEN/Akt pathway, mediating primordial follicle activation under chemotherapy. Supportively, when administered to female rodents under cyclophosphamide treatment, AS101 was found to reduce activation and subsequent exhaustion of ovarian quiescent follicles, thus preserving fertility (20) without compromising the effectiveness of antineoplastic treatment (40).

G-CSF: Interestingly, Granulocyte colony stimulating factors, frequently used against chemotherapy induced myelotoxicity, can maintain ovarian function in mice models under treatment alkylating factors, by promotion of neovascularization of the ovarian tissue (41), what may protect the ovaries from chemotherapy related ischemia.

Tamoxifen: Tamoxifen, an estrogen antagonist used in hormone-dependent breast cancer, has been also explored as a potential fertility preservation agent. As it has been shown in rodent studies, co-administration of tamoxifen with cyclophosphamide and doxorubicin seems to preserve ovarian follicle deposit (42). Although the underlying mechanism has not been clarified, it has been suggested that tamoxifen upregulates IGF-1 (Insulin-like Growth Factor 1), which protects primordial follicles from oxidative stress (7).

## Conclusion—Further Questions

Although a variety of alternatives in order to maintain ovarian function and fertility in female cancer survivors, diagnosed and undergoing chemotherapy at a young age, adequately established practices to do so are lacking. Notably, study of the applicable literature reveals a relative lack of clinical evidence regarding preservation of patient fertility among a variety of malignancies mostly affecting children, adolescents, and young adults of both genders, such as CNS tumors, germ cell neoplasms, osseous and soft tissue sarcomas. Similarly, fertility preservation in young patients affected by cancer types more frequent in older ages, such as early stage colon cancer, has not been investigated sufficiently either.

Although current oncofertility guidelines are universal among different tumor types and patient profiles (43), potential disparities between patients due to age, chemotherapy agents employed, and the malignancy itself may also interfere with fertility preservation practices. Consequently, a more methodical investigation of fertility preservation strategies, considering the above parameters, is required, in order to adequately establish the most efficient practices for each patient group.

Especially regarding young female cancer survivors, in an era that age of pregnancy is pushed even after the age of 40, it is of major importance to further investigate and collect sufficient evidence, aiming to safely guide patients and physicians in everyday clinical practice. Until then, oncologists should not neglect this domain of life of their female, younger patients; female cancer patients have to be encouraged to express their concerns and wishes, regarding fertility and pregnancy after antineoplastic treatment completion, in order to organize a plan of action that will allow them to maintain a normal endocrine function as well as the possibility to create a family.

## Author Contributions

All authors contributed to the article and approved the submitted version.

## Conflict of Interest

The authors declare that the research was conducted in the absence of any commercial or financial relationships that could be construed as a potential conflict of interest.
